# Effectiveness of focused extracorporeal shock wave therapy in the treatment of carpal tunnel syndrome: A randomized, placebo-controlled pilot study

**DOI:** 10.1007/s00508-020-01785-9

**Published:** 2020-12-22

**Authors:** Christina Gesslbauer, Michael Mickel, Othmar Schuhfried, Dominikus Huber, Mohammad Keilani, Richard Crevenna

**Affiliations:** Department of Physical Medicine, Rehabilitation and Occupational Medicine, Medical University of Vienna, Währinger Gürtel 18–20, 1090 Vienna, Austria

**Keywords:** Pain management, Conservative management, Median neuropathy, Night splint, Treatment outcome

## Abstract

**Background:**

The carpal tunnel syndrome is the most common entrapment neuropathy in the general population. A conservative treatment should be considered in mild to moderate cases. The aim of this study was to assess the effect of a focused extracorporeal shock wave therapy in the treatment of mild to moderate carpal tunnel syndrome.

**Material and Methods:**

In this study 30 patients were randomly assigned into 2 groups. Subjects in the study group received three sessions of focused extracorporeal shock wave therapy, whereas the control group underwent a sham therapy. Patients were evaluated 3 and 12 weeks after treatment. The primary outcome was the visual analogue scale score. Secondary outcome measurements included hand grip strength, Boston Carpal Tunnel Syndrome Questionnaire, SF-36 Health Survey and electrodiagnostic measurements.

**Results:**

A significant improvement of visual analogue scale at week 3 (*p* = 0.018) and week 12 (*p* = 0.007) as well as hand grip strength at week 12 (*p* = 0.019) could be observed in the study group. The study group showed a significantly better sensory nerve conduction velocity at week 12 than the control group, before correcting for multiple testing, and also a significant improvement in distal motor latency of the median nerve at week 12 (*p* = 0.009) as well as in both questionnaires (SF-36 subscale bodily pain, *p* = 0.020 and severity symptom scale, *p* = 0.003). No such improvement was observed in the control group.

**Conclusion:**

Focused extracorporeal shock wave therapy is an effective and noninvasive treatment method for mild to moderate carpal tunnel syndrome.

## Introduction

The carpal tunnel syndrome (CTS) is the most common entrapment neuropathy in the general population. It is a condition caused by compression of the median nerve. Most cases of CTS are idiopathic leading to chronic pressure increase and subsequently ischemia of the median nerve and segmental demyelination [1]. The CTS is more common among women as compared to men. Further risk factors include repetitive wrist movements, pregnancy, obesity, rheumatoid arthritis, diabetes mellitus and menopause. Symptoms usually start gradually at night and include paresthesia, burning and/or tingling in the territory of the median nerve as well as nocturnal pain and in severe cases also weakness of the hand and thenar atrophy [1]. In chronic and untreated cases CTS can lead to irreversible nerve damage. The diagnosis of CTS is usually clinical in a patient with characteristic symptoms and includes a physical examination and electrodiagnostic testing. There are different methods to treat CTS patients [1, 2]. A conservative treatment should be considered in mild to moderate cases and consists of splinting, physical modalities (e.g. therapeutic ultrasound, low level laser therapy), oral corticosteroids or nonsteroidal anti-inflammatory drugs. Other treatment options include steroid injection and surgery. In severe cases a surgical treatment should be considered [1–5]. Positive effects especially for wrist splint, local corticosteroid injection and surgical treatment have been demonstrated in multiple studies [1–5]; however, steroid injection and surgery always carry some risks of side effects (e.g. infections or allergic reactions). Extracorporeal shock wave therapy (ESWT) is a noninvasive and novel treatment option. It is based on the generation of acoustic waves which interact directly with cells by mechanotransduction by activating the metabolic rate which leads to tissue remodelling [6]. The ESWT can be classified into focused ESWT (fESWT) and radial ESWT (rESWT). While rESWT has a more superficial effect and reaches the maximum energy at the skin surface and distributes it radially into the tissue, fESWT develops the maximum energy at a focus located deeper in the body tissues [7]. Over the last years ESWT has gained widespread attention. Different types of diseases and conditions can be treated in a very effective and safe way without severe side effects [8, 9]. It has been demonstrated to have anti-inflammatory, analgesic and proliferative effects [10–15] and it has also been proven to have effects for the reinnervation of peripheral nerves [16–18]. Therefore, both fESWT and rESWT, have received increased attention in the treatment of CTS and several studies have already yielded partially positive effects [19–23]. Xu et al. demonstrated significantly greater improvement in visual analogue scale (VAS) and the Boston carpal tunnel syndrome questionnaire (BCTQ) in the ESWT group compared to the local corticosteroid injection group [19]. Wu et al. reported positive results after a treatment with rEWST. They concluded that rESWT is a safe and effective method for relieving pain [20]. Similar effects were shown in a study of Vahatpour et al. [21]. Because ESWT is a novel treatment there is still little known about the efficacy, the long-term effects and adverse events of ESWT in the treatment of CTS. Hence, the aim of this study was to assess the effect of fESWT in the treatment of mild to moderate carpal tunnel syndrome.

## Materials and Methods

### Trial design and participants

This pilot study was a randomized, single-blinded, placebo-controlled pilot study.

The project was approved by the ethics committee of the Medical University of Vienna (EK Nr. 1080/2019) and all subjects gave written informed consent to participate. The study conformed to the principles of the Declaration of Helsinki. There was no commercial sponsorship.

Inclusion criteria were mild to moderate CTS objectively verified using electrodiagnostic testing [24, 25]. Exclusion criteria were metabolic diseases, blood clotting disorders, systemic diseases, polyneuropathy, chemotherapy during the study, corticosteroid therapy, use of anticoagulation, history of trauma/surgery or nerve lesions of the treated extremity, CTS surgery on the affected hand, implantable cardioverter defibrillator (ICD)/pacemaker implantation, other therapy for the affected hand during the study, acute inflammation or infections, severe mental illnesses/psychiatric diseases, and severe neurological diseases.

Patients from the outpatient clinic (Department of Physical Medicine, Rehabilitation and Occupational Medicine) who met the inclusion criteria and who were diagnosed with mild to moderate CTS by electrodiagnostic testing were randomized to receive either fESWT or sham fESWT. A total of 30 patients were enrolled, all of which participated in the determination of the baseline measurements. Of these 20 patients proceeded to receive treatment and the follow-up examinations, 4 of the patients dropped out for personal reasons, while the remaining 6 drop-outs could not complete the trial due to coronavirus disease 2019 (COVID-19) restrictions on visits to the clinic. The results were analyzed on a per protocol basis.

In the study group (fESWT group), participants received weekly fESWT for 3 consecutive weeks. In the control group the participants received sham fESWT for the same interval. Additionally, all subjects were asked to wear night splints. Outcome measure were the VAS [26], hand grip strength (using a Jamar hand grip dynamometer [JAMAR® dynamometry (Patterson Medical, Warrenville, IL, USA)] [27]) and electrodiagnostic parameters (distal motor latency and sensory nerve conduction velocity) [24, 25]. Subjects were also tested with questionnaires by using the SF-36 Health Survey (SF-36) [28] and the BCTQ [29, 30]. All patients were evaluated at baseline (T0: VAS, hand grip, electrodiagnostic parameters, questionnaires), week 3 (T1: VAS, hand grip, questionnaires), and week 12 (T2: VAS, hand grip, electrodiagnostic parameters, questionnaires) after treatment by the same physician. Of the patients 20 have completed the 3‑month evaluation. No adverse effects occurred.

### Randomization and blinding

Randomization was performed by using sequentially numbered sealed envelopes. Eligible participants were then randomly assigned to either the fESWT group (intervention group) or the control group (sham fESWT). The participants and the investigator who evaluated the baseline/outcome measures were blinded with respect to the group allocation.

### Intervention

**Fig. 1:**
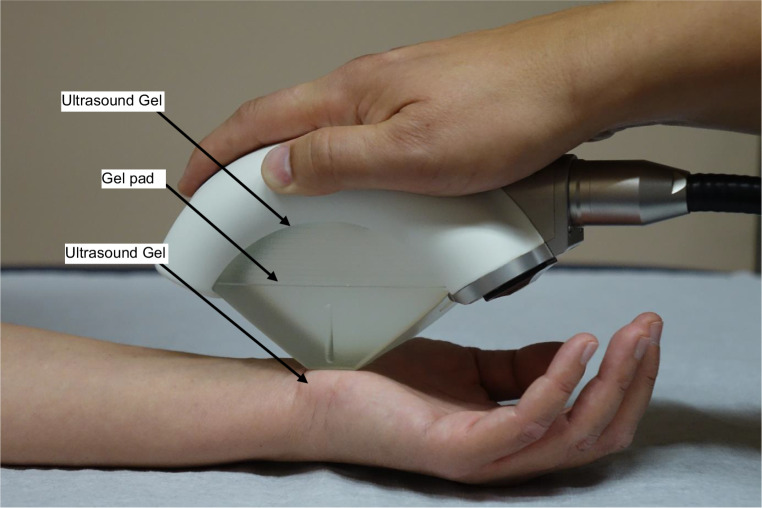
Application of focused extracorporeal shock wave therapy

Patients in the fESWT group were treated three times with fESWT (PiezoWave2, Richard Wolf GmbH, Knittlingen, Germany) (Fig. 1). After identifying the carpal tunnel area by using musculoskeletal ultrasonography (ECUBE i7, Alpinion Medical Systems, Seoul, Korea) fESWT was applied to the flexor retinaculum (transverse carpal ligament) by using a linear therapy source. Each patient received a linear fESWT (therapy source FBL 10X5G2, PiezoWave2) that comprised 500 shocks at energy flux density (EFD) of 0.05 mJ/mm^2^ (maximum). The pulse repetition frequency was 4 Hz. In the fESWT group, a three-layered coupling medium between the applicator head and the tissue/skin was used (ultrasound gel-gel pad-ultrasound gel) to efficiently transduce the shock wave into the tissue (Fig. 1). In the control group only a two-layered coupling medium was used and the ultrasound gel layer between applicator head and gel pad was omitted to achieve a placebo treatment effect. The treatment process was identical to that of the fESWT group, with the same sound signals during the procedure. Since there is still no standardized treatment recommendation of fESWT for CTS we chose to perform the fESWT/sham fESWT once a week for a period of 3 weeks by the same physician. Additionally, all subjects were asked to wear night splints.

### Outcome measures

All patients were evaluated at baseline (T0: VAS, hand grip, electrodiagnostic parameters, questionnaires) week 3 (T1: VAS, hand grip, questionnaires), and week 12 (T2: VAS, hand grip, electrodiagnostic parameters, questionnaires).

#### Primary outcome measure

1. The subjective pain intensity was measured with a 100 mm VAS, 0 indicating no pain and 100 the strongest imaginable pain [25].

#### Secondary outcomes

2. The distal motor latency of the median nerve (DML) and antidromic sensory conduction velocity of the median nerve (SNCV) were evaluated by using NCV (Keypoint device, Medtronic, Dantec Medical A/S, Skovlunde, Denmark) [24, 25]. The ulnar nerve was also evaluated to exclude other peripheral neuropathies. All evaluations were executed in the same room by the same physiatrist.
3. The hand grip strength was evaluated using a hand grip dynamometer [28].
4. The 36-item Short Form Health Survey questionnaire (SF-36) consists of 36-items and 8 scales and measures the physical and mental health [29].
5. The BCTQ evaluates the severity of symptoms and the functional status of patients with carpal tunnel syndrome. The symptom severity scale (SSS) consists of 11 questions covering symptom severity and scores from 1 point (mildest) to 5 points (most severe). The functional status scale (FSS) consists of 8 points ranging from 1 point (no difficulty with the activity) to 5 points (cannot perform the activity at all) [29, 30].

### Statistical analysis

Statistical analysis was carried out using GraphPad Prism 8 software (GraphPad, San Diego, CA, USA).

Testing for normality was assessed groupwise via the Shapiro-Wilk test. When employing an ANOVA, sphericity was not assumed and therefore not tested for. No significant outliers in the data analyzed via ANOVA were observed. Sample size was computed with a power of 0.80.

A *p‑value* < 0.05 was considered as statistically significant. The 95% confidence interval for mean differences is reported in brackets next to the value.

If not specified otherwise, the Holm-Sidak method for correcting for multiple testing was applied to all pairwise comparisons and adjusted *p*-values reported accordingly.

## Results

### Patients

A total of 20 patients completed the 3month follow-up. Table 1 shows the baseline demographic data and clinical characteristics of patients who completed the study. The mean age in the fESWT group (*n* = 10) was 55.8 ± 4.6 years. They experienced CTS symptoms for a mean of 29.0 ± 32.8 months.

**Table 1:** Demographic data for both drop-outs and included groups. A Holm–Sidak test showed no significant difference between these groups regarding the demographics

	fESWT group		Control group		Drop-outs	
	Mean	SD	Mean	SD	Mean	SD
Age (years)	55.80	4.66	54	17.40	53.10	7.48
Female sex, *n* (%)	8	80%	6	60%	7	70%
Height (cm)	167.80	6.61	169.70	11.81	171.40	11.07
Weight (kg)	81.10	18.91	87.40	29.72	71.30	7.53
Hoffman–Tinel sign, *n* (%)	1.40	0.52	1.30	0.48	0.80	0.42
Duration of symptoms (months)	29	32.89	33.60	44.26	40.80	40.77

*fESWT* focused extracorporeal shock wave therapy, *SD* standard deviation

The mean age in the control group (*n* = 10) was 54.0 ± 17.4 years. They experienced CTS symptoms for a mean of 33.6 ± 44.2 months.

*T*‑tests for multiple comparisons between the fESWT, the control, and the drop-out group for all main outcome variables showed no significant differences in baseline measurements (Tables 2, 3 and 4). Table 5 gives an overview of all outcome measures at each point in time.

**Table 2:** Main outcome parameters fESWT vs. control group at T0

	*P-*value	Mean of fESWT group	Mean of control group	Difference	SE of difference	t ratio	Adjusted *p-* value
VAS	0.49	50.5	58.3	−7.80	10.96	0.71	0.98
Hand grip (kg)	0.54	29.9	27.3	2.60	4.15	0.63	0.98
DML (ms)	0.58	4.88	4.76	0.12	0.21	0.56	0.98
SNCV (m/s)	0.18	40.2	38.35	1.85	1.32	1.41	0.74
SSS	0.92	2.41	2.44	−0.04	0.34	0.11	>0.99
FSS	0.88	1.73	1.79	−0.06	0.42	0.15	>0.99
SF-36 PAIN	0.95	47.9	48.4	−0.50	8.60	0.06	>0.99

*VAS* Visual analogue scale, *DML *Distal motor latency, *SNCV* Sensory nerve conduction velocity, *SSS* Symptom severity scale, *FSS* Functional status scale, *SF-36* PAIN Short Form 36 Health Survey, *fESWT* focused extracorporeal shock wave therapy, *SE* standard error

**Table 3:** Main outcome parameters fESWT vs. drop-out group at T0

	*P* value	Mean of fESWT group	Mean of drop-outs	Difference	SE of difference	t ratio	Adjusted *p-* value
VAS	0.76	50.5	47.5	3.00	9.83	0.31	0.98
Hand grip (kg)	0.70	29.9	28.6	1.30	3.36	0.39	0.98
DML (ms)	0.63	4.88	4.989	−0.11	0.22	0.49	0.98
SNCV (m/s)	0.34	40.2	72.89	−32.69	33.38	0.98	0.88
SSS	0.95	2.409	2.431	−0.02	0.39	0.06	0.98
FSS	0.19	1.725	2.172	−0.45	0.32	1.38	0.76
SF-36 PAIN	0.21	47.9	36.8	11.10	8.57	1.30	0.76

*VAS* Visual analogue scale, *DML* Distal motor latency, *SNCV* Sensory nerve conduction velocity, *SSS* Symptom severity scale, *FSS* Functional status scale, *SF-36 PAIN* Short Form 36 Health Survey, *fESWT *focused extracorporeal shock wave therapy, *SE* standard error

**Table 4:** Main outcome parameters control vs. drop-out group at T0

	*P-*value	Mean of control group	Mean of drop-outs	Difference	SE of difference	t ratio	Adjusted *p-*value
VAS	0.35	58.3	47.50	10.80	11.21	0.96	0.85
Hand grip (kg)	0.72	27.3	28.60	−1.30	3.54	0.37	0.92
DML (ms)	0.20	4.76	4.99	−0.23	0.17	1.34	0.73
SNCV (m/s)	0.32	38.35	72.89	−34.54	33.36	1.04	0.85
SSS	0.98	2.445	2.43	0.01	0.46	0.03	0.98
FSS	0.40	1.788	2.17	−0.38	0.45	0.86	0.85
SF-36 PAIN	0.15	48.4	36.80	11.60	7.63	1.52	0.67

*VAS* Visual analogue scale, *DML* Distal motor latency, *SNCV* Sensory nerve conduction velocity, *SSS* Symptom severity scale, *FSS* Functional status scale, *SF-36 PAIN* Short Form 36 Health Survey, *fESWT *focused extracorporeal shock wave therapy, *SE* standard error

**Table 5:** Mean, standard deviation, and confidence interval of the mean for all outcome variables at each point of measurement

	fESWT group					Control group				
	Mean	Std. deviation	Std. error of mean	Lower 95% CI of mean	Upper 95% CI of mean	Mean	Std. deviation	Std. error of mean	Lower 95% CI of mean	Upper 95% CI of mean
VAS T0	51	21	6.7	35	66	58	27	8.6	39	78
VAS T1	31	25	7.8	13	48	42	25	7.8	24	59
VAS T2	28	23	7.4	11	44	46	24	7.7	28	63
Hand grip T0 (kg)	30	8.9	2.8	23	36	27	9.6	3	20	34
Hand grip T1 (kg)	32	10	3.2	24	39	29	6.7	2.1	24	34
Hand grip T2 (kg)	34	9.7	3.1	27	41	28	8	2.5	22	33
DML T0 (ms)	4.9	0.57	0.18	4.5	5.3	4.8	0.37	0.12	4.5	5
DML T2 (ms)	4.5	0.64	0.2	4.1	5	4.5	0.6	0.19	4.1	5
SNCV T0 (m/s)	40	3.9	1.2	37	43	38	1.5	0.46	37	39
SNCV T2 (m/s)	44	5.7	1.8	40	48	39	2.8	0.9	37	41
SF-36 PAIN T0	48	21	6.7	33	63	48	17	5.4	36	61
SF-36 PAIN T1	58	14	4.4	48	68	44	23	7.2	28	60
SF-36 PAIN T2	54	24	7.5	37	71	47	22	7.1	31	63
SSS T0	2.4	0.58	0.18	2	2.8	2.4	0.9	0.28	1.8	3.1
SSS T1	1.7	0.36	0.12	1.4	1.9	2.1	0.86	0.27	1.5	2.7
SSS T2	1.9	0.95	0.3	1.2	2.6	1.8	0.67	0.21	1.3	2.3
FSS T0	1.7	0.71	0.23	1.2	2.2	1.8	1.1	0.36	0.98	2.6
FSS T1	1.5	0.6	0.19	1	1.9	1.6	0.7	0.22	1.1	2.1
FSS T2	1.6	0.8	0.25	0.97	2.1	1.7	0.82	0.26	1.1	2.2

*VAS* Visual analogue scale, *DML* Distal motor latency, *SNCV* Sensory nerve conduction velocity, *SSS* Symptom severity scale, *FSS* Functional status scale, *SF-36 PAIN* Short Form 36 Health Survey, *fESWT *focused extracorporeal shock wave therapy, *SE* standard error, *Std. deviation* standard deviation, *CI* confidence interval

### Intergroup comparison

Multiple *t*‑tests showed no statistically significant difference between the fESWT and the control group at T1 (Table 6). At T2, the fESWT group did significantly better regarding SNCV (mean difference = 5.0, SE of difference = 2.0, *p* = 0.02) before correcting for multiple testing (Table 7). After correction via the Holm-Sidak method, however, there was no significant difference between groups.

**Table 6:** Differences in outcome measures at T1 corrected via the Holm–Sidak method for multiple testing

T1	*P-*value	Mean of fESWT group	Mean of control group	Difference	SE of difference	t ratio	Adjusted *p-*value
VAS	0.34	30.6	41.5	−10.90	11.02	0.99	0.71
Hand grip (kg)	0.5	31.6	29	2.60	3.80	0.69	0.75
SSS	0.15	1.66	2.11	−0.45	0.30	1.51	0.48
FSS	0.58	1.46	1.63	−0.16	0.29	0.56	0.75
SF-36 PAIN	0.12	58.1	44.3	13.80	8.37	1.65	0.46

*VAS* Visual analogue scale, *SSS* Symptom severity scale, *FSS* Functional status scale, *SF-36 PAIN* Short Form 36 Health Survey, *fESWT* focused extracorporeal shock wave therapy, *SE* standard error

**Table 7:** Differences in outcome measures at T2 corrected via the Holm–Sidak method for multiple testing

T2	*P-*value	Mean of fESWT group	Mean of control group	Difference	SE of difference	t ratio	Adjusted *p-*value
VAS	0.1	27.5	45.8	−18.30	10.64	1.72	0.48
Hand grip (kg)	0.14	33.8	27.6	6.20	3.98	1.56	0.52
DML (ms)	0.94	4.52	4.54	−0.02	0.28	0.07	0.98
SNCV (m/s)	0.02	43.75	38.75	5.00	2.01	2.48	0.15
SSS	0.71	1.93	1.79	0.14	0.37	0.37	0.98
FSS	0.76	1.55	1.66	−0.11	0.36	0.31	0.98
SF-36 PAIN	0.49	53.9	46.6	7.30	10.28	0.71	0.93

*VAS* Visual analogue scale, *DML* Distal motor latency, *SNCV* Sensory nerve conduction velocity, *SSS* Symptom severity scale, *FSS* Functional status scale, *SF-36 PAIN* Short Form 36 Health Survey, *fESWT *focused extracorporeal shock wave therapy, *SE* standard error

### ANOVA by time and group

#### VAS

**Fig. 2:**
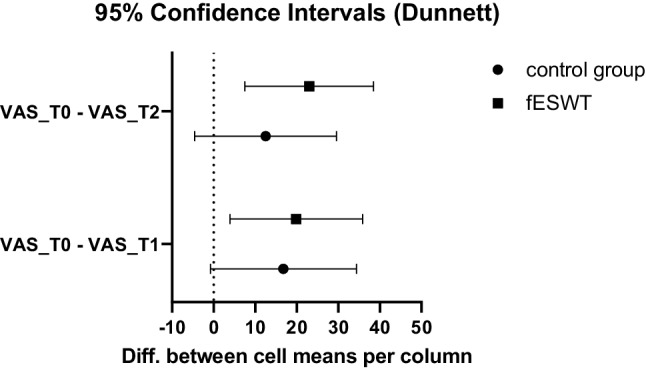
Confidence intervals for difference in pain (cell means) between baseline (T0) vs. posttreatment (T1) (bottom) and baseline vs. 3‑month follow-up (T2) (top). There is a significant improvement in pain only in the verum group. Despite a visible trend in T2 vs. T0, no difference could be detected between the two treatment groups (columns)

In absolute values, VAS decreased over time in the fESWT group. The control group showed no clear direction of VAS over time (Fig. 2). A two-way repeated measurements ANOVA for pain (VAS) at T0, T1 and T2 for both groups showed a significant effect of time (*p* < 0.001) and no significant effect of treatment group (*p* = 0.216) or the interaction of the two (*p* = 0.477). Multiple comparisons corrected by Dunnet’s test revealed a significant difference of VAS between both T0 and T1 (mean difference = 19.9, 95% confidence interval [3.93; 35.9], *p* = 0.018) and T0 and T2 (mean difference = 23.0, 95% confidence interval [7.51; 38.5], *p* = 0.007) in the fESWT group (Table 8; Fig. 2). In the control group, no significant difference could be observed (mean difference T0 vs. T1 = 16.8, 95% confidence interval [−0.761; 34.4], *p* = 0.059; mean difference T0 vs. T2 = 12.5, 95% confidence interval [−4.56; 29.6], *p* = 0.151).

**Table 8:** Pair-wise comparison of all outcome parameters after carrying out a repeated-measures two-way ANOVA (Analysis of variance) for the three time points T0, T1 and T2 for the respective variable

Outcome parameter/test	Mean diff	95.% CI of diff	Adjusted *p-*value
*(Dunnet’s)*			
*Control group*			
VAS_T0 vs. VAS_T1	16.8	−0.76 to 34.36	0.0599
VAS_T0 vs. VAS_T2	12.5	−4.56 to 29.56	0.1504
*fESWT group*			
VAS_T0 vs. VAS_T1	19.9	3.92 to 35.87	0.018
VAS_T0 vs. VAS_T2	23	7.51 to 38.49	0.0069
*(Dunnet’s)*			
*Control group*			
Hand-grip strength T0 vs. T1	−1.7	−6.65 to 3.25	0.5893
Hand-grip strength T0 vs. T2	−0.3	−3.98 to 3.38	0.9673
*fESWT group*			
Hand-grip strength T0 vs. T1	−1.7	−4.46 to 1.06	0.2363
Hand-grip strength T0 vs. T2	−3.9	−7.08 to −0.72	0.0194
*(Tukey’s)*			
*Control group*			
DML T0 vs. SNCV T0	−33.59	−35.25 to −31.93	<0.0001
DML T0 vs. T2	0.22	−0.08 to 0.52	0.1666
DML T0 vs. SNCV T2	−33.99	−37.07 to −30.91	<0.0001
SNCV T0 vs. DML T2	33.81	31.90 to 35.72	<0.0001
SNCV T0 vs. T2	−0.4	−2.56 to 1.76	0.9358
DML T2 vs. SNCV T2	−34.21	−37.52 to −30.90	<0.0001
*fESWT group*			
DML T0 vs. SNCV T0	−35.32	−39.34 to −31.30	<0.0001
DML T0 vs. T2	0.36	0.09 to 0.62	0.0094
DML T0 vs. SNCV T2	−38.87	−44.80 to −32.94	<0.0001
SNCV T0 vs. DML T2	35.68	31.57 to 39.79	<0.0001
SNCV T0 vs. T2	−3.55	−7.55 to 0.45	0.0847
DML T2 vs. SNCV T2	−39.23	−45.29 to −33.17	<0.0001
*(Dunnet’s)*			
*fESWT group*			
SF36 pain T0 vs. T1	−10.2	−18.59 to −1.81	0.0204
SF36 pain T0 vs. T2	−6	−20.88 to 8.89	0.4954
*Control group*			
SF36 pain T0 vs. T1	4.1	−3.95 to 12.15	0.349
SF36 pain T0 vs. T2	1.8	−6.96 to 10.56	0.8169
*(Dunnet’s)*			
*fESWT group*			
SSS T0 vs. SSS T1	0.7454	0.31 to 1.18	0.0028
SSS T0 vs. SSS T2	0.4819	−0.09 to 1.06	0.1016
*Control group*			
SSS T0 vs. SSS T1	0.3365	−0.45 to 1.13	0.4636
SSS T0 vs. SSS T2	0.6547	0.02 to 1.33	0.0551

*VAS* Visual analogue scale, *DML* Distal motor latency, *SNCV* Sensory nerve conduction velocity, *SSS* Symptom severity scale, *FSS* Functional status scale, *SF-36 PAIN* Short Form 36 Health Survey, *fESWT *focused extracorporeal shock wave therapy, *SE* standard error, *CI* confidence interval

### Hand grip

**Fig. 3:**
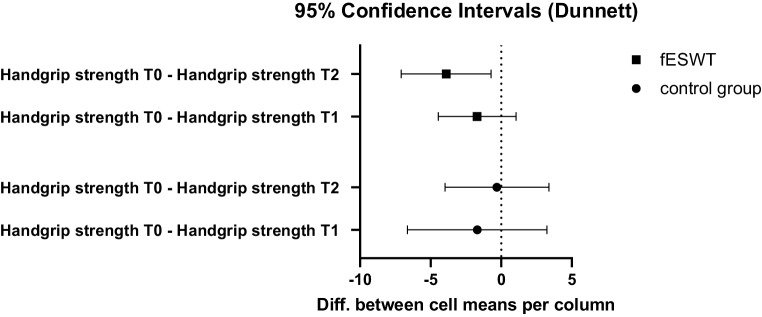
Differences in hand grip strength at T0 vs. T1 and T0 vs. T2 in both fESWT and control group showed a tendency of improvement

The two-way repeated measurements ANOVA for hand grip strength at T0, T1 and T2 yielded no significant effect of any kind (time: *p* = 0.108; group: *p* = 0.330; time * group: *p* = 0.140) [The interaction term time * group describes the effect we are interested in, i.e. the effect on patients in the fESWT (group) group after intervention (time)]. Dunnet’s multiple comparisons test, however, revealed a significant improvement on baseline hand grip strength at T2 in the fESWT group (mean difference T2 vs. T0 = 3.90 [0.72; 7.08], *p* = 0.019) (Table 8; Fig. 3).

### Electroneurography

Due to the naturally high correlation of these variables, a two-way repeated-measurements ANOVA was carried out for SNCV and DML simultaneously. The analysis of variance yielded significant effects of time (*p* < 0.001), treatment group (*p* = 0.024) and their interaction (*p* = 0.010). Corrected via Tukey’s test for multiple comparisons, a significant improvement of DML (mean difference= −0.36, 95% confidence interval [−0.62; −0.10], *p* = 0.009) but no significant effect for sNCV (mean difference= 3.55, 95% confidence interval [−0.450; 7.55], *p* = 0.084) was observed in the fESWT group (Table 8).

### SF-36

The SF-36 subscale bodily pain was compared at all three points of measurement T0, T1, and T2 via two-way repeated measurements ANOVA. Time (*p* = 0.515) and treatment group (*p* = 0.431) showed no significant effect on pain scoring but their interaction did (*p* = 0.049). Dunnet’s test for multiple comparisons showed a significant effect on the SF-36 pain score at T0 compared to T1 in the fESWT group (mean difference= −10.2, 95% confidence interval [−18.6; −1.81], *P* = 0.020) (Table 8), but no other significant results.

### Boston carpal tunnel syndrome questionnaire

**Fig. 4:**
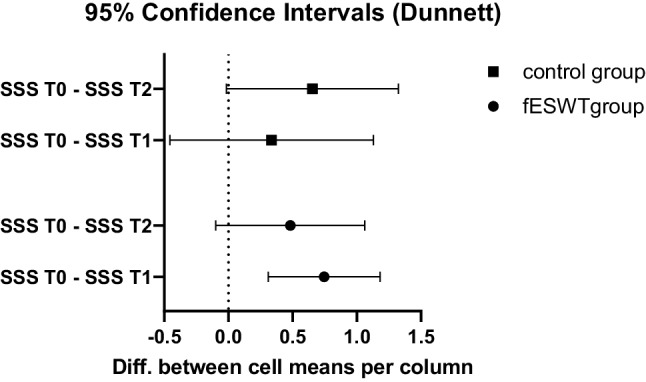
Differences in Boston carpal tunnel syndrome questionnaire (symptom severity scale) at T0 vs. T1 and T0 vs. T2 showed a significant short-term improvement in the fESWT group

A two-way repeated-measurements ANOVA at T0, T1 and T2 for the SSS suggested a significant effect of time (*p* = 0.002), while treatment group (*p* = 0.681) and time * group (*p* = 0.213) showed no significant difference. A pairwise comparison using Dunnet’s test revealed a significant difference between SSS at T0 and T1 (mean difference= 0.74, 95% confidence interval [0.31; 1.18], *p* = 0.003) but no other differences (Table 8; Fig. 4). Generally, participants in both groups saw a small increase of their scores over time.

## Discussion

The aim of this randomized, single blinded, placebo-controlled pilot study was to investigate the effect of three sessions of fESWT in patients with mild to moderate CTS. Our results strengthen the therapeutic effects of (f)ESWT once more. Compared to the control group, a significant improvement of VAS in the fEWST group appeared after three sessions of fESWT. The trend in the follow-up after 3 months showed a further significant improvement of VAS and a significant improvement of hand grip strength in the fESWT group. Concerning electrodiagnostic parameters, our study found a significant decrease of DML in the fESWT group at week 12 compared to baseline while the other electrodiagnostic parameter (sNCV) did not show any significant improvement between baseline and week 12 although there was a greater difference in the fESWT group for sNCV compared to the control group. Considering the SF36 questionnaire, improvement was observed for the subscale bodily pain at week 12 in the fESWT group. These results are in accordance with the results of VAS.

The CTS is the most common form of median nerve entrapment. In mild to moderate cases a conservative treatment should be considered. The use of ESWT for CTS is a novel treatment method and growing data have shown that ESWT is an effective and potential treatment option [11, 12, 14, 19–23]. Seok et al. administered one session of fESWT to treat patients with CTS and compared them to patients after corticosteroid injection. They found significant reductions in VAS and BCTQ scores in both groups at 1 and 3 months follow-up. Mild improvement was noted in the fESWT group for nerve conduction parameters. The authors concluded that ESWT could be as useful as corticosteroid injections [14]. Paoloni et al. reported that patients with mild to moderate CTS might experience pain relief and increased functionality after three sessions of fESWT compared to US and cyro ultrasound therapy [12]. In a study of Wu et al. the intervention group received three session of rESWT with night splinting, whereas the control group received sham rESWT and night splinting. A significantly greater improvement in VAS, BCTQ and cross-sectional area (CSA) of the median nerve was shown in the intervention group. They concluded that rESWT is an effective method for pain relief [20]; however, the exact biological effects of ESWT still remain unknown and the definitive mechanism of ESWT on peripheral nerves/neuropathy is unclear. It has been demonstrated that ESWT seems to stimulate the production of endothelial nitric oxide (NO), angiogenesis and neurogenesis through vascular endothelial growth factor (VEGF), has anti-inflammatory effects through reduction in the release of calcitonin gene-related peptide (CGRP) and has also neuronal regeneration effects and promotes axonal regeneration of peripheral nerves through molecular reactions [13, 31–34]. Shock waves also stimulate tenocyte proliferation and collagen synthesis [35, 36]. In 2001 Ohtori et al. concluded that shock wave application to rat skin causes reinnervation of sensory nerve fibers [16]. Hausner et al. investigated whether ESWT improves the regeneration of injured nerves in an experimental rat model and proved that ESWT is effective in promoting axonal regeneration [17]. Mense et al. reported similar findings [18]. Very interesting are also the findings of Miyamoto et al. who compared the elasticity and thickness of the transverse carpal ligament by sonoelastography. Their results showed that the transverse carpal ligament was significantly thicker and harder in CTS patients and concluded that an increased stiffness could be one of the reasons for CTS [37]. In summary the anti-inflammatory, antinociceptive, neuronal regeneration effects as well as the apparent influence of the extracellular matrix might be the main mechanism for CTS improvement when using fESWT. It is possible that the anti-inflammatory effect can reduce the perineural pressure and neuronal regeneration may lead to an improvement of the electrodiagnostic parameters. Moreover, we hypothesize that the treatment with linear focused shock waves may stimulate the elasticity of the transverse carpal ligament via mechanotransduction effects and therefore relieve the CTS symptoms. Hence, it would be interesting for future studies to investigate if morphological changes of the median nerve also occur during/after fESWT.

There were some limitations of this study, most importantly the low number of participants and the high number of drop-outs. This limitation was largely due to the sudden closure of facilities due to COVID-19. Although no significant differences in baseline measurements between the drop-outs and any of the groups could be observed, the per protocol analysis and power could be improved in future studies. Furthermore, our findings indicate that the long-term results at both 3 months and longer intervals of follow-up examinations deserve a closer look when assessing the efficacy of ESWT.

## Conclusion

Our results suggest that fESWT is an effective and noninvasive treatment method for mild to moderate carpal tunnel syndrome. Further studies with larger sample size and longer follow-up period are needed to verify the clinical efficacy of fESWT.
